# Unexpected *CEP290* mRNA Splicing in a Humanized Knock-In Mouse Model for Leber Congenital Amaurosis

**DOI:** 10.1371/journal.pone.0079369

**Published:** 2013-11-06

**Authors:** Alejandro Garanto, Sylvia E. C. van Beersum, Theo A. Peters, Ronald Roepman, Frans P. M. Cremers, Rob W. J. Collin

**Affiliations:** 1 Department of Human Genetics, Radboud University Medical Centre, Nijmegen, The Netherlands; 2 Institute for Genetic and Metabolic Disease, Radboud University Medical Centre, Nijmegen, The Netherlands; 3 Nijmegen Centre for Molecular Life Sciences, Radboud University Medical Centre, Nijmegen, The Netherlands; 4 Department of Otorhinolaryngology, Head and Neck Surgery, Radboud University Medical Centre, Nijmegen, The Netherlands; 5 Donders Institute for Brain, Cognition and Behaviour, Radboud University Medical Centre, Nijmegen, The Netherlands; National Eye Institute, United States of America

## Abstract

Leber congenital amaurosis (LCA) is the most severe form of retinal dystrophy with an onset in the first year of life. The most frequent genetic cause of LCA, accounting for up to 15% of all LCA cases in Europe and North-America, is a mutation (c.2991+1655AG) in intron 26 of *CEP290*. This mutation generates a cryptic splice donor site resulting in the insertion of an aberrant exon (exon X) containing a premature stop codon to *CEP290* mRNA. In order to study the pathophysiology of the intronic *CEP290* mutation, we generated two humanized knock-in mouse models each carrying ~6.3 kb of the human *CEP290* gene, either with or without the intronic mutation. Transcriptional characterization of these mouse models revealed an unexpected splice pattern of *CEP290* mRNA, especially in the retina. In both models, a new cryptic exon (coined exon Y) was identified in ~5 to 12% of all *Cep290* transcripts. This exon Y was expressed in all murine tissues analyzed but not detected in human retina or fibroblasts of LCA patients. In addition, exon x that is characteristic of LCA in humans, was expressed at only very low levels in the retina of the LCA mouse model. Western blot and immunohistochemical analyses did not reveal any differences between the two transgenic models and wild-type mice. Together, our results show clear differences in the recognition of splice sites between mice and humans, and emphasize that care is warranted when generating animal models for human genetic diseases caused by splice mutations.

## Introduction

Leber congenital amaurosis (LCA; OMIM 204000) is a group of rare and severe inherited retinal dystrophies with a prevalence of ~1:50,000 individuals worldwide [1,2]. The clinical characteristics of LCA include severe and early vision loss that appears in the first year of life, amaurotic pupils, sensory nystagmus and the absence of electrical signals on electroretinogram (ERG) [3]. Like other retinal disorders such as retinitis pigmentosa (RP; OMIM 268000), LCA shows a high genetic heterogeneity. Currently, mutations in 19 different genes have been identified (RetNet: https://sph.uth.edu/retnet), mainly segregating in an autosomal recessive manner. The most frequently mutated LCA gene is *CEP290* that encodes the centrosomal protein 290 kDa [3,4]. The most prevalent LCA-causing mutation in *CEP290*, accounting for up to 15% of LCA cases in many Western countries, is a deep-intronic change (c.2991+1655AG; p.C998*) that generates a strong splice donor site which results in the insertion of a cryptic exon with a premature stop codon into ~50% of *CEP290* transcripts [4-6].

To date, more than one hundred mutations have been identified in *CEP290* [4] (CEP290 homepage - Eye diseases – LOVD: http://grenada.lumc.nl/LOVD2/eye/home.php?select_db=CEP290), causing a wide spectrum of phenotypes ranging from isolated retinal dystrophy (LCA, early-onset RP) [5,7] to more severe syndromes such as Senior-Løken syndrome (SLS; OMIM 266900) [8], Joubert syndrome (JS; OMIM 213300) [9], Bardet-Biedl syndrome (BBS; OMIM 209900) [10] and Meckel-Grüber syndrome (MKS; OMIM 249000) [11]. Unfortunately, no clear phenotype-genotype correlation has been established yet. The prevalent intronic change, however, is predominantly found in individuals with non-syndromic retinal dystrophy and is considered to be a hypomorphic mutation.

The *CEP290* gene was first isolated from human brain cDNA libraries, and encompasses 54 exons that encode a 2479 amino acid protein [12]. CEP290 has been localized to the centrosome and to the transition zone of cilia [13,14]. The exact physiological role of CEP290 remains unclear, although it has been shown that it plays an important role in the regulation of ciliary protein trafficking and cilium assembly [13,15]. In photoreceptor cells, CEP290 localizes to the connecting cilium [16], similar to approximately one third of proteins encoded by retinal dystrophy genes [17]. The connecting cilium is the transition zone of the photoreceptor sensory cilium, connecting the inner and the outer segment of rods and cones, the light-sensitive neurons responsible of the conversion of light stimuli into chemical signals that are transmitted to the brain [18]. 

Two naturally occurring mutant *Cep290* animal models have been described to date. The murine *rd16* model carries a genomic deletion encompassing exons 35-39 of *Cep290*, that results in an in-frame deletion of 897 bp in *Cep290* mRNA. These mice show an early, fast and progressive photoreceptor degeneration, where only one row of photoreceptor cell nuclei is detected at postnatal day 30 (P30) [16]. In a subpopulation of Abyssinian cats with retinal degeneration, an intronic mutation (c.6960+9TG) has been identified. This mutation generates a new strong canonical splice donor site that inserts four nucleotides into *CEP290* mRNA, causing a frameshift that results in a premature stop codon after two amino acids [19]. The first clinical symptoms appear at the age of seven months when reduced electroretinogram recordings are detected as a result of rod degeneration that is followed by cone death, leading to complete blindness at the age of 3-5 years [20]. 

In order to study and understand the pathophysiology of the intronic *CEP290* mutation c.2991+1655AG, we have generated two humanized mouse models, in which human exon 26, intron 26 (with or without the LCA mutation) and exon 27 were inserted into the murine *Cep290* gene via homologous recombination. A detailed characterization of these mice at the transcriptional level revealed unexpected splicing of *CEP290* mRNA, that was only partially in line with the aberrant splicing observed in patients with *CEP290*-associated LCA. Our data suggest species-specific differences in the splicing machinery between mice and humans, indicating that mice may not always represent suitable models to study the pathogenic mechanisms underlying genetic variants that affect splicing. 

## Materials and Methods

### Ethics statement

All procedures were performed after obtaining approval from the Radboud University Nijmegen ethics committee for experimental animal research (RU-DEC-2012-023), and according to the regulations of the ARVO statement for the use of animals in ophthalmic and vision research. For human skin biopsies written informed consent was gathered from all individuals in Clinical Genetics Center Nijmegen by signing the declaration of permission for the use of body material (*Toestemmingsverklaring gebruik lichaamsmateriaal*) of the Radboud University Medical Center and our research followed the tenets of the Declaration of Helsinki. All procedures were carried out in The Netherlands.

### Generation of the transgenic models

Two animal models were generated by GenOway (Lyon, France). Briefly, the targeting vector contained the human genomic region between introns 25 and 27, either without (*Cep290* ^*hum/hum*^) or with the c.2991+1655AG mutation (*Cep290* ^*lca/lca*^). Neomycine (NEO) and Diphtheria Toxin A (DTA) cassettes were also subcloned for positive and negative selection, respectively. The final vector was electroporated in 129Sv/Pas ES cells. A total of 260 clones were positively selected. After validating the homologous recombination event at the 5’- and the 3’-end by PCR and southern blotting, four ES cell clones for *Cep290* ^*hum/hum*^ and one for *Cep290* ^*lca/lca*^ were isolated. These clones were injected in blastocysts from C57BL/6J females and injected in pseudo-pregnant females of the same strain. Based on the colour, four highly chimeric males were selected for breeding with Cre deleter females in order to excise the NEO cassette for each model. Excision was validated by PCR and Southern blot. The resulting heterozygous animals were used to obtain homozygous offspring. Wild-type C57BL/6J mice (*Cep290* ^*wt/wt*^) were used as a reference in all analyses.

### Animal handling, tissue dissection and preparation of the samples

Tissue samples were obtained from postnatal day 150 (P150) *Cep290* ^*wt/wt*^, *Cep290* ^*hum/hum*^ and *Cep290* ^*lca/lca*^ models. Animals were euthanized with CO_2_ followed by cervical dislocation. Retina, brain, kidney, liver, spleen, testis and lung were dissected and immediately frozen in liquid nitrogen for RNA and protein isolation, whereas for cryosectioning, eyes were enucleated, embedded in OCT (Optical Cutting Temperature compound, Tissue-Tek, Sakura Finetek, Torrance, CA) and frozen in isopentane and liquid nitrogen. From each of the three models, two animals were used for RNA isolation, two for immunohystochemistry and four for protein analysis.

### Cell culture of human fibroblast cell lines

Fibroblast cell lines derived from skin biopsies of individuals with *CEP290*-associated LCA or healthy controls, were cultured in DMEM, supplemented with 20% fetal bovine serum, 1% penicillin-streptomycin and 1% of sodium pyruvate at 37°C and 5% CO_2_. Informed consent was gathered from all individuals and our research followed the tenets of the Declaration of Helsinki. 

### RNA isolation

Several tissues from P150 *Cep290* ^*wt/wt*^, *Cep290* ^*hum/hum*^ and *Cep290* ^*lca/lca*^ mice were obtained for transcriptional analysis. Twenty milligrams of tissue (brain, kidney, liver, spleen, testis and lung) and a pool of two retinas from different animals of the three different mouse models were used for RNA isolation (Nucleospin RNA II, Düren, Germany). For fibroblast cell lines, RNA was isolated from ~1.5 million cells by using the same kit following the manufacturer’s protocol. Human adult retina RNA was purchased from Clontech (Mountain View, CA).

### RT-PCR and transcriptional analysis

One microgram of RNA (from tissue, fibroblasts or human retina) was used for cDNA synthesis by using the iScript cDNA Synthesis kit (Bio-Rad, Hercules, CA) at a final volume of 20 µl and then diluted by adding 50 µl of RNAse-free H_2_O. For tissue expression analysis, all reaction mixtures (25 µl) contained 10 µM of each primer pair, 2 µM of dNTPs, 1.5 mM MgCl_2_, 10% Q-solution (Qiagen, Venlo, Netherlands), 1 U of Taq polymerase (Roche, Penzberg, Germany) and 5 µl of diluted cDNA. PCR conditions were 94°C for 2 min, followed by 35 cycles of 20 s at 94°C, 30 s at 58°C and 30 s at 72°C, with a final extension step of 2 min at 72°C. Amplicons were analyzed by agarose electrophoresis. Semi-quantification was performed using Image J software [21]. Actin expression was used to compare and normalize the samples, primers for actin amplification were designed to amplify both the human and the mouse gene. Oligonucleotide sequences are listed in Table S1.

### Western blot analysis

A pool of two retinas of different animals from each mouse model were homogenized in 200 µl of RIPA buffer (50 mM Tris pH 7.5, 1 mM EDTA, 150 mM NaCl, 0.5% Na-Deoxycholate, 1% NP40 plus protease inhibitors). Protein quantification was performed with a BCA kit (Thermo Fisher Scientific, Waltham, MA). For CEP290 detection, ~170 µg of total protein lysate supplemented with sample buffer was loaded onto a NuPage 3-8% tris-acetate gel (Life technologies, Carlsbad, CA). The electrophoresis was carried out for 4 h at 150 V. For normalization with an anti-α-tubulin antibody, ~30 µg of the same protein lysates were loaded onto a NuPage 4-12% bis-acrylamide tris-glycine gel (Life technologies, Carlsbad, CA) and run for 2 h at 150 V. A protein lysate from HEK293-T cells overexpressing FLAG-tagged CEP290 was used as a positive control (40 µg and 10 µg were loaded for CEP290 and tubulin detection, respectively). All lysates were boiled for 5 min at 98°C prior to loading. Proteins were transferred to a PVDF membrane (GE Healthcare, Little Chalfont, UK) overnight at 25 V at 4°C. Blots were blocked in 5% non-fat milk in PBS, incubated overnight at 4°C with rabbit anti-CEP290 (dilution 1:750, Novus Biological, Littleton, CO) or mouse anti-α-tubulin (dilution 1:2000, Abcam, Cambridge, UK) in 0.5% non-fat milk in PBS, washed in PBST (4 x 5 min), incubated with the appropriate secondary antibodies for 1 h at room temperature (RT), washed in PBST (4 x 5 min) and developed using the Odissey Imaging System (Li-Cor Biosciences, Lincoln, NE). Western blot analysis was performed in duplo. Semi-quantification was performed using Image J software [21]. 

### Immunohistochemistry

Seven micrometer cryosections were dried for 1 h at RT, washed in PBS to remove the OCT, permeabilized for 20 min in 0.01% Tween in PBS and blocked for 30 min (0.1% ovalbumin and 0.5% fish gelatine in PBS). Primary antibody incubation was performed overnight at 4°C by diluting the antibodies in blocking solution. The next day, sections were washed in PBS (3 x 10 min), incubated for 45 min at RT with the corresponding Alexa fluor-conjugated secondary antibodies and DAPI, washed in PBS (3 x 10 min) and mounted using Prolong Gold antifade kit (Life Technologies, Carlsbad, CA). The antibodies and dilutions used were: 1:300 for rabbit anti-CEP290 (Novus Biological, Littleton, CO), 1:1000 for mouse anti-acetylated tubulin (Sigma-Aldrich, St. Louis, MO) and 1:500 for secondary antibodies Alexa Fluor 488 and 568 raised in goat against rabbit and mouse IgGs, respectively (Molecular Probes, Eugene, OR).

## Results

### Generation of the humanized models

**Figure 1:**
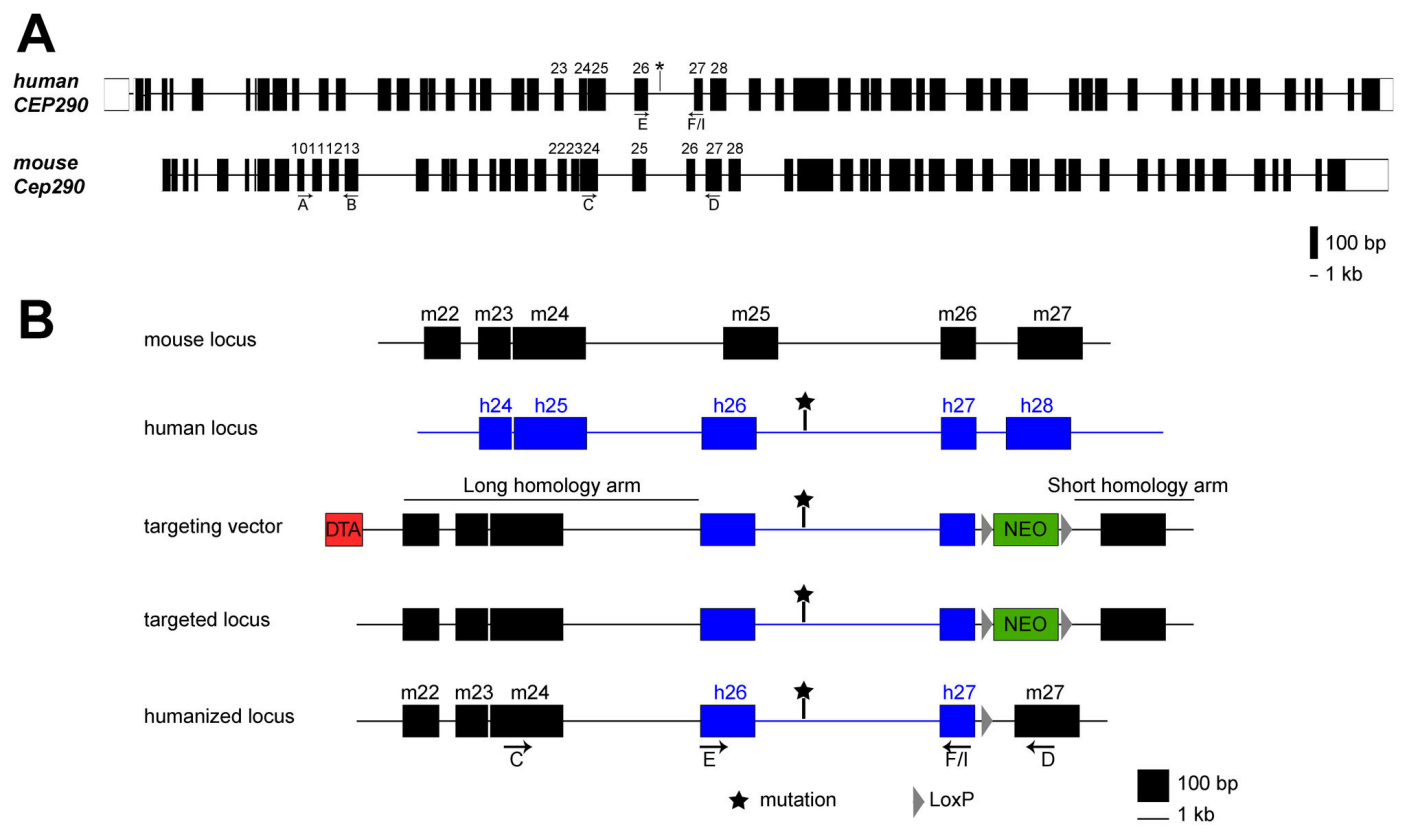
Generation of the *Cep290* humanized mouse models. (A) Structure of the *CEP290* gene in human and mouse (drawn to scale). (B) Two mouse models were generated by introducing human exons 26 and 27 and intron 26, either with or without the LCA-causing mutation (depicted with *), to the mouse *Cep290* gene. Human and mouse loci, targeting vector and recombinant locus are depicted. Arrows and letters indicate the position of the oligonucleotides (see Table S1).

The murine *Cep290* gene contains 53 exons (one less than in humans) and shares a high homology at DNA (86%) and protein (87% homology and 94% similarity) levels with human *CEP290* (Figure 1A). In order to generate a humanized knock-in mouse model that would mimic the genotype and phenotype associated with the deep-intronic LCA-causing mutation, a recombination strategy was designed that consisted of the replacement of mouse exons 25 (mE25) and 26 (mE26), as well as intron 25 (mI25), by the human counterparts (hE26, hI26/hI26mut and hE27) (Figure 1B). All recombination events were validated by PCR and Southern blot analysis (data not shown). The isolated positive ES-cell clones were injected in blastocysts that were implanted in pseudo-pregnant females. Highly chimeric males were bred with Cre deleter females to excise the NEO cassette. The resulting heterozygous mice were used to obtain homozygous animals, which were viable and fertile. Subsequent breedings were done between homozygous animals. 

### Transcriptional analysis of *Cep290* in the humanized models

**Figure 2:**
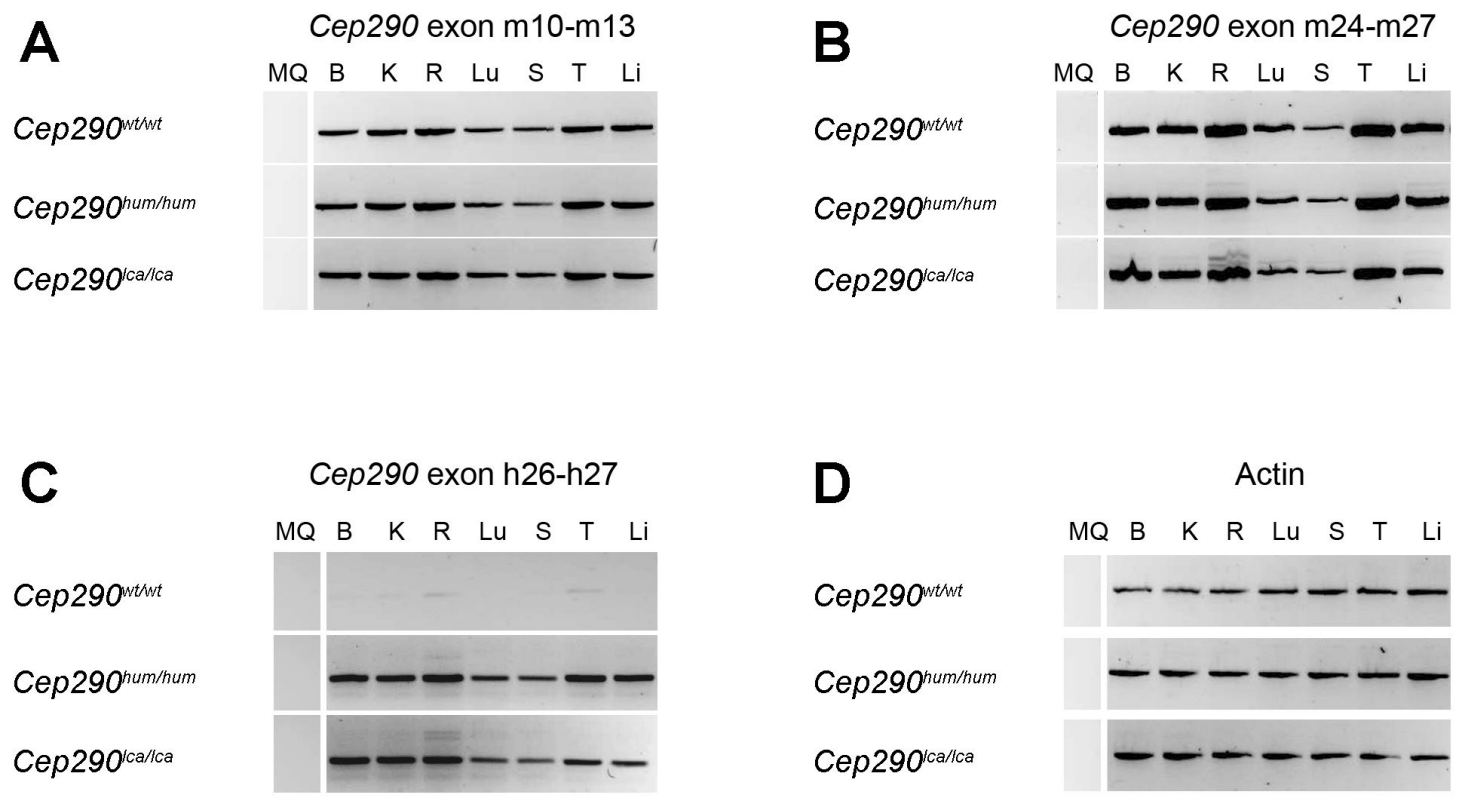
Transcriptional characterisation of *Cep290* in humanized mouse models. (A-B) *Cep290* expression levels in various mouse tissues were assessed by RT-PCR. Regions containing murine exons 10 to 13 (A) and 24 to 27 (B) were analyzed. No differences were observed. (C) Amplification using human primers was also assessed in the three models. Only humanized models showed amplification. (D) Actin was used for normalization and comparison among tissues and models. MQ: H_2_O (negative control); B: Brain; K: Kidney; R: Retina; Lu: Lung; S: Spleen; T: Testis and Li: Liver.

To determine whether the humanization of the murine *Cep290* would compromise the general *Cep290* gene expression, we determined by RT-PCR the transcriptional levels of *Cep290* in two different regions of *Cep290* cDNA, using actin expression as a reference (Figure 2). First, we amplified the region from exon 10 to exon 13 (Figure 2A), which is not affected by the recombination. In addition, we performed a PCR from mouse exons 24 to 27, which encompasses the humanized region (hE26-hE27) instead of mE25 and mE26 (Figure 2B). In both cases, no differences in the amount of *Cep290* transcripts were observed between the two models (*Cep290* ^*lca/lca*^ and *Cep290* ^*hum/hum*^) and the wild-type mice (*Cep290* ^*wt/wt*^), indicating that the human exons did not alter the expression levels of *Cep290* in mouse. Intriguingly however, especially in the retina, additional bands were detected in both the *Cep290* ^*hum/hum*^ and the *Cep290* ^*lca/lca*^ model but not in wild-type mice. In lymphoblastoid cell lines derived from LCA patients homozygously carrying the intronic *CEP290* mutation, two different *CEP290* transcripts are detected in equal ratios, the correctly spliced product and an aberrant one, that contains the 128-bp cryptic exon X that introduces a premature stop codon [22]. In the *Cep290* ^lca/lca^ model, some minor bands were detected, that could represent transcripts which include the 128-bp-exon X. However, additional bands were also observed in the *Cep290* ^*hum/hum*^ mice (Figure 2B), suggesting new unexpected splice variants caused by the humanization of the murine *Cep290* locus. 

To investigate these aberrant splicing events in more detail, we used primers that were located in the human exons (hE26 and hE27). Due to the high homology of these two exons between both species (E26: 93% and E27: 87%), we designed the primers in the regions with the lowest sequence homology to ensure amplification of only the human exon. As expected, the two humanized models, but not the wild-type mice, showed robust amplification of the human exons. However, also in wild-type mice, some products were detected in the tissues where *Cep290* is most highly expressed, such as retina, kidney or testis, likely due to the high similarity between the human and mouse *CEP290* exons (Figure 2C). In line with the results found using primers in mE24 and mE27, again, aberrant splice products were detected in the retinas of both humanized mouse models (Figure 2C).

### Expression of CEP290 cryptic exons in humans and mice

**Figure 3:**
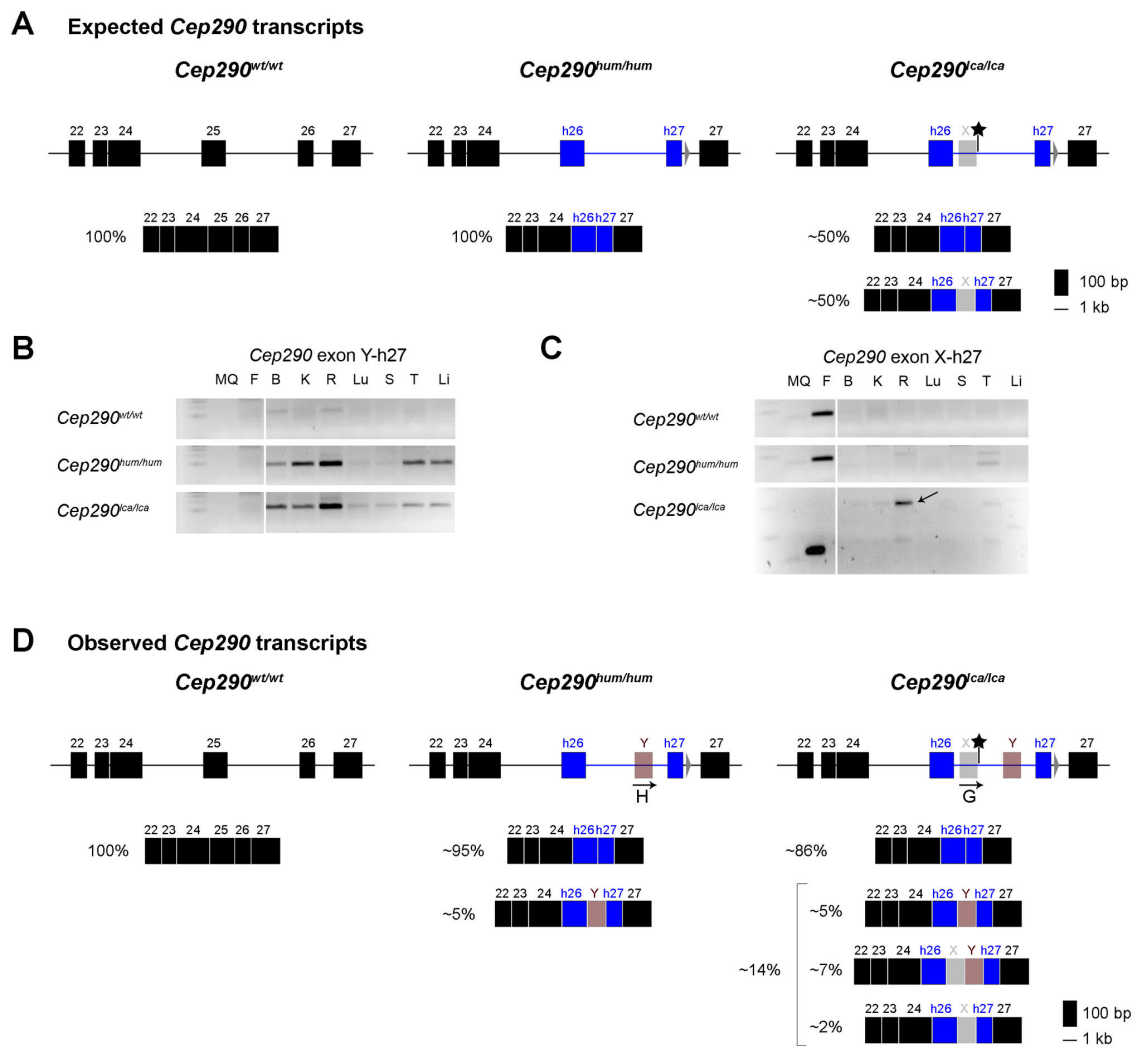
Analysis of the *CEP290* cryptic exons. (A) Expected transcripts for each model. (B) Exon Y is expressed in all tissues in either *Cep290* ^*hum/hum*^ and *Cep290* ^*lca/lca*^ mice. (C) Only in the *Cep290* ^*lca/lca*^ model, exon X is expressed, either with exon Y or without, at very low levels. The arrow indicates the band that contains both cryptic exons (exon X and exon Y) in the same transcript. *Cep290* ^lca/lca^ PCR products were run longer to clearly separate the two different bands. (D) Schematic representation of the different transcripts containing cryptic exons found in the three mouse models. Mouse exons and introns are depicted in black, while human exons and introns are represented in blue. Cryptic exons X and Y are shown in grey and red, respectively. Arrows and letters indicate the position of the oligonucleotides in the cryptic exons. Semi-quantification was performed using ImageJ software [21]. MQ: H_2_O (negative control); F: human LCA fibroblasts; B: Brain; K: Kidney; R: Retina; Lu: Lung; S: Spleen; T: Testis and Li: Liver.

**Figure 4:**
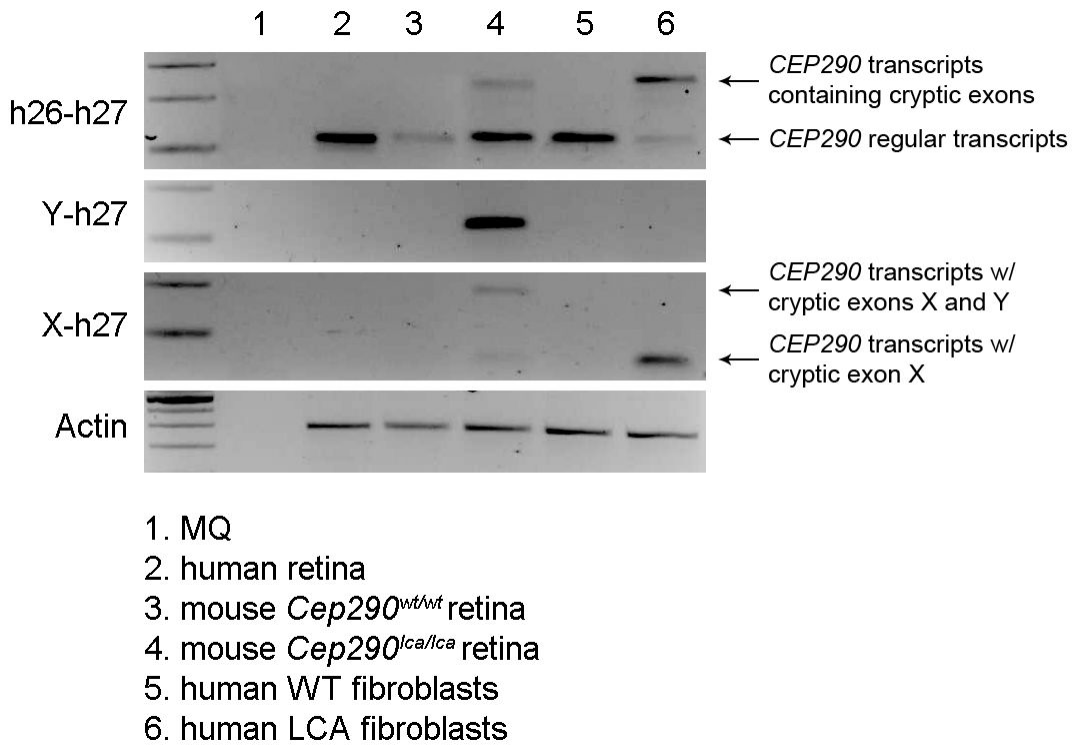
Analysis of the cryptic exons X and Y in human retina and fibroblast cell lines. The expression of the *CEP290* human exons 26 and 27, and the cryptic exons Y and exon X were assessed in tissues derived from both mice and humans. Actin was used for normalization.

The fact that the *Cep290* ^hum/hum^ model also showed evidence of aberrant *Cep290* splicing, whereas only one transcript was expected (Figure 3A), prompted us to study the nature of these aberrant splice products in depth. Sanger sequencing analysis revealed the presence of a new exon of 116 bp (coined exon Y), which was found to be also located in human intron 26, downstream of exon X (starting position exon Y: c.2992-1603). RT-PCR analysis using a primer located in exon Y allowed us to observe that *Cep290* transcripts encoding exon Y were expressed in almost all tissues, but most prominent in the retina (Figure 3B), both in the *Cep290* ^*hum/hum*^ and in the *Cep290* ^*lca/lca*^ model. When using a primer located in exon X, two bands were observed derived from the retina of *Cep290* ^*lca/lca*^ mice (Figure 3C). Sequence analysis revealed that the most prominent band corresponded to a *Cep290* transcript containing both exons X and Y. *Cep290* transcripts that contained only exon X were also found, but expressed at very low levels that were hardly detectable. In total, we were able to identify one transcript in the *Cep290* ^*wt/wt*^ animals, two in the *Cep290* ^*hum/hum*^ and four in the *Cep290* ^*lca/lca*^ model (Figure 3D). Semi-quantification of all aberrant splicing events revealed that in the *Cep290* ^*hum/hum*^ model, approximately 5% of *Cep290* transcripts contained exon Y, whereas in the *Cep290* ^*lca/lca*^ model, ~14% of all *Cep290* transcripts contained aberrant exons, i.e. exon Y, exon X or both (Figure 3D). Following the identification of these unexpected splicing events, we investigated whether exon Y is a naturally occurring exon in human tissues. For that purpose, RNA from human retina as well as from fibroblast cells from healthy and LCA (homozygously carrying the *CEP290* intronic mutation) individuals were used, together with retinal RNA from the *Cep290* ^wt/wt^ and *Cep290* ^*lca/lca*^ mice. RT-PCR analysis revealed that transcripts containing exon Y were not detected in healthy retinas nor in fibroblasts derived from individuals with *CEP290*-associated LCA (Figure 4). Moreover, we observed again that the transcripts containing exon X are less abundant than those containing exon Y in *Cep290* ^lca/lca^ retinas (Figure 4). 

### Western blot and immunohistochemical analysis of mouse retinas

**Figure 5:**
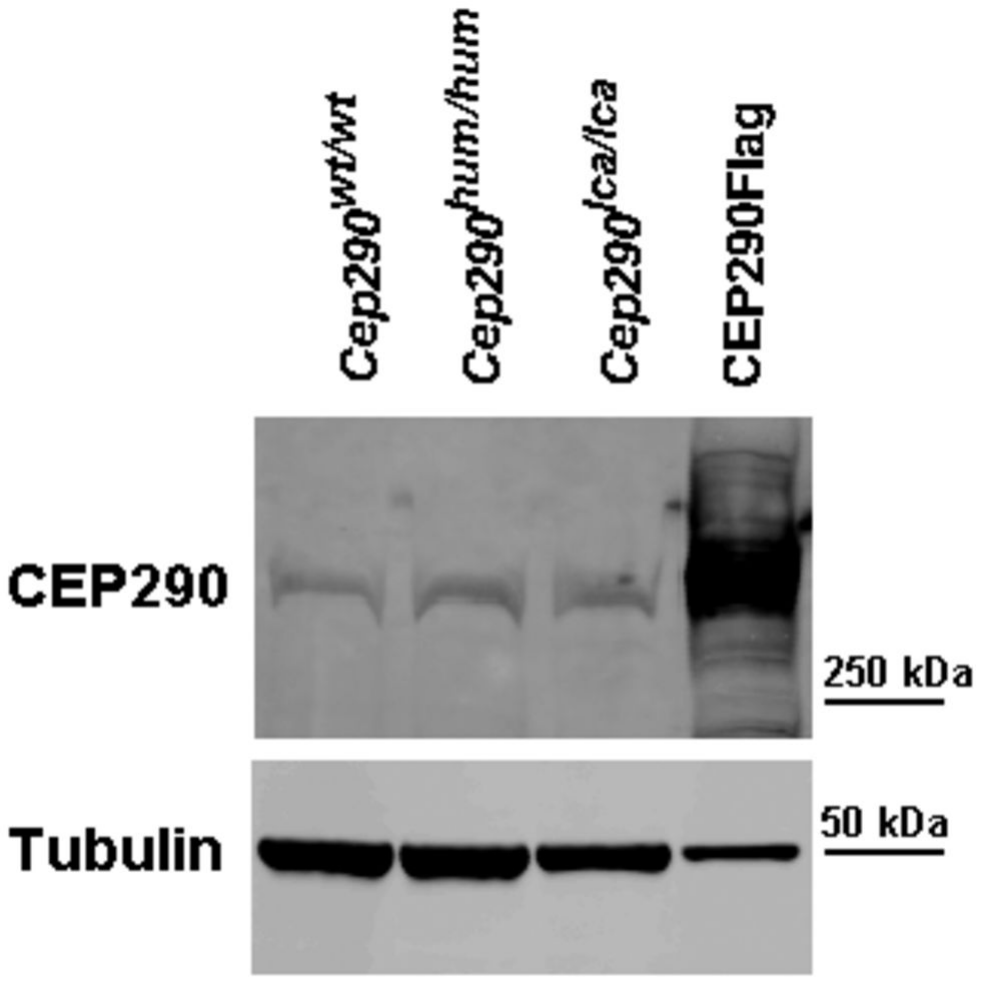
CEP290 protein analysis in *Cep290* mouse models. Representative CEP290 immunodetection in P150 retinas from the three different mouse models. A CEP290-Flag construct was expressed in HEK293-T cells and used as a positive control. Tubulin was used for normalization.

The insertion of exon X, exon Y, or both, to the regular *Cep290* transcript, in all cases is predicted to cause a frame-shift and premature termination of the Cep290 protein. To determine the effect of these various splicing events on the amount of CEP290 protein levels, Western blot analysis was performed using retinal protein lysates from our mouse models. Large amounts (>150 µg) of total protein were required to robustly detect CEP290. Western blot analysis revealed no significant differences among the three models (Figure 5), indicating that the inclusion of any or both cryptic exons to only a small percentage of *Cep290* transcripts does not significantly alter Cep290 protein levels.

**Figure 6:**
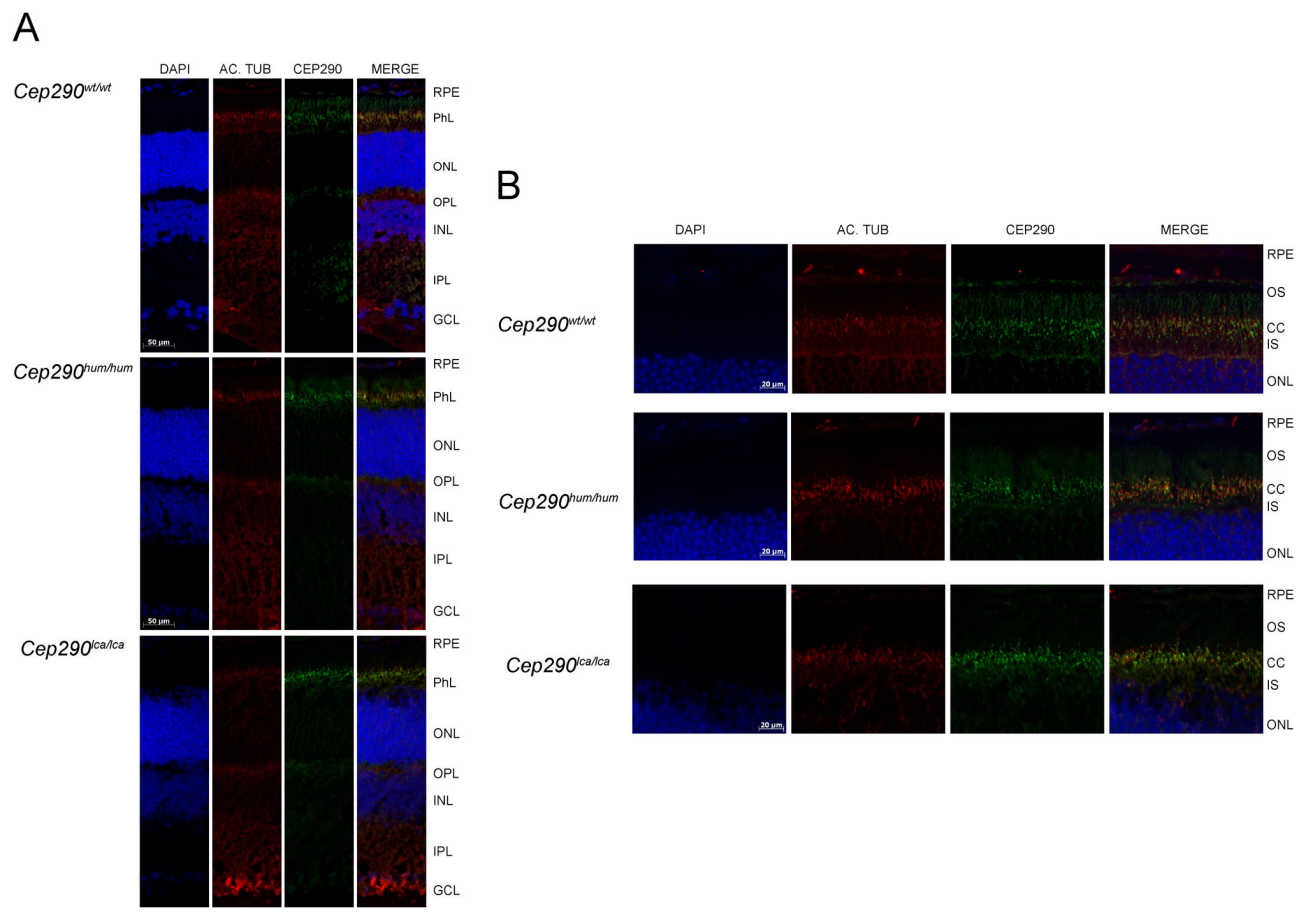
Morphological and CEP290 immunolocalization analyses in P150 mouse retinas. Immunodetection of CEP290 and acetylated tubulin in retinal sections from *Cep290* ^*wt/wt*^, *Cep290* ^*hum/hum*^ and *Cep290* ^*lca/lca*^ mice, at P150. Images were taken at 200X (A) and 400x (B) magnifications. CEP290 (green) is located in the connecting cilium (CC) and remains unaltered in the humanized models. Nuclei were marked with DAPI (blue) and CC with acetylated tubulin (red). GCL: ganglion cell layer; IPL: inner plexiform layer; INL: inner nuclear layer; OPL: outer plexiform layer; ONL: outer nuclear layer; PhL: photoreceptor layer; RPE: retinal pigment epithelium; IS: inner segment; CC: connecting cilium; OS: outer segment.

Finally, retinal sections from *Cep290* ^*wt/wt*^, *Cep290* ^hum/hum^ and *Cep290* ^lca/lca^ mice at P150 were analyzed by immunohistochemistry. The overall morphology of all retinas was similar, as were the amounts of photoreceptor cell nuclei in the different models. In addition, CEP290 was correctly localized at the connecting cilium of photoreceptors in all three models (Figure 6), illustrating that the low abundance of aberrant *Cep290* splicing also does not appear to affect the structural integrity of the retina. 

## Discussion

Rodents, in particular mice, have been widely used in the last decades to shed light on the pathophysiology of several diseases as well as for the development of therapeutic trials. In the field of vision science, they are particularly useful because of their relatively short lifespan, which allows a quick read-out of the progression of retinal degeneration. In addition, there are several naturally occurring retinal degeneration models [23,24], the interactions between retinal cells are similar in humans and mice [25], and more importantly, the genetic manipulation and subsequent phenotypic characterization are very well established. 

Here, we generated a transgenic *CEP290* knock-in model by introducing the deep intronic mutation that causes LCA in humans [5] and its flanking exons to elucidate the mechanisms that cause photoreceptor cell death in these patients. In addition, mimicking the splice abnormalities that are observed in LCA patients in a mice model would allow to determine the efficacy of therapeutic interventions, like for instance antisense oligonucleotide-based rescue of aberrant *CEP290* splicing that was shown to be effective in patient cell lines by us and others [22,26]. The high homology between human and mouse exons allowed the generation of the animal model without changing the open reading frame of *Cep290* and only slightly altering the amino acid composition of CEP290. In addition, the naturally occurring *Cep290* mouse model (*rd16*) shows a clear early-onset retinal phenotype, indicating an essential role for this gene in the mouse retina [16]. As expected, the expression levels of the *Cep290* gene were not altered in our humanized mouse models, with highest expression shown in retina and testis. However, when the composition of *Cep290* transcripts was analyzed in detail, we did not detect the same ratio of correctly and aberrantly spliced transcripts (approximately 1:1) as detected in cell lines of LCA patients carrying the intronic *CEP290* mutation [22]. Instead, unexpected aberrant splicing events were detected, that included the recognition of a novel exon Y, in addition to exon X. In total, in the *Cep290* ^*lca/lca*^ model, four different transcripts were observed, including two that contained the aberrant exon X associated with LCA (Figure 3D). Nevertheless, the amount of all aberrantly spliced transcripts collectively was less than 15% of the total *Cep290* expression, which is most probably not enough to compromise retinal function. This is further supported by the absence of significant differences in CEP290 protein levels between the models assessed by Western blot analysis, and of any abnormalities in retinal structure following (immuno)histochemical analysis of retinal sections.

In the past, others have successfully used humanized knock-in mouse models to mimic the pathophysiological mechanisms underlying splice site mutations for several inherited diseases [27-30]. In this study, even though the humanization of mouse *Cep290* was not compromising the gene expression in general, the splicing machinery of the mouse appeared not to be able to recognize the human splice acceptor site or the splice donor site that is generated by the intronic *CEP290* mutation, suggesting differential recognition of splice sites in humans *vs.* mice. In addition, the substitution of the murine intron by the human one generated another cryptic exon, robustly expressed in the mouse retina, but not found in human retinas nor in fibroblasts. Again, these results point to differences in the splicing machineries in mice *vs.* humans, as it has already been suggested for other retinal dystrophy genes [31]. One example is *CERKL*, which shows a high transcriptional complexity in human and mouse retina, showing different alternative splice events between species in addition to the use of alternative promoters [31]. In addition, it has been showed that the recognition of splice sites are species-, regulator region- and RNA-structure-dependent [32], suggesting that the human exon X might have an inaccessible structure for the murine spliceosome or that some enhancers favor the insertion of exon Y in mice. 

In the last decade, the use of other non-murine animal models has been regularly preferred to study retinal dystrophies, for a number of reasons. It is well known that like in humans, primate and raptor retinas contain a cone-enriched zone called fovea, whereas in mice the cones are homogeneously distributed throughout the retina [18], thus less reminiscent of the human situation. In addition, several genes in which mutations are causative for retinal dystrophy in humans do not have an orthologue in rodent species, like for instance *EYS* [33,34]. Other mutant mouse models do not show any phenotype, potentially because of functional redundancy. For example, the GCAP1 (*GUCA1A*) and GCAP2 (*GUCA1B*) proteins are guanylate cyclases activators involved in phototransduction. Although in humans, mutations in either of these genes can cause retinal dystrophy, mice only show a phenotype when both genes are knocked out at the same time [35,36]. Occasionally, the protein might not be localized in the expected cell type, as is the case with the aforementioned *CERKL* gene, which in mouse is mainly expressed in ganglion cells rather than in photoreceptors [37]. Other genes that, when mutated in mice, do not resemble the human phenotypes are *USH2A* and *RDH12*. In *Ush2a* ^*-/-*^ mice retinal degeneration only appears at very late stages [38], whereas in *Rdh12* knock-out mice, no retinal phenotype is observed at all [39]. Taken together, these examples suggest that proteins involved in visual function might not play the same roles or are not involved in the same critical functions in humans *vs.* mice. The question that now arises is which is the best model to study retinal dystrophies. Although there are models with anatomically more similar eyes (i.e. dogs or primates), in these medium- and large-sized animal models, genetic manipulation is practically impossible due to experimental, financial and ethical restrictions. In the past, other naturally occurring animal models have enabled the study of the retinal degeneration and the start of the development of new therapies, such as *RPE65* gene augmentation therapy in dogs [40]. However, compared to mice, the disease is only manifested after several years and also the progression is slower.

In summary, we have generated and characterized a humanized transgenic mouse model that carries the prevalent intronic *Cep290* mutation causative for LCA. Despite the recognition of expected and unexpected cryptic exons, the total amount of aberrantly spliced *Cep290* transcripts was less than 15%, and thereby does not mimic the aberrant *CEP290* splicing observed in individuals with *CEP290*-associated LCA. Our results indicate that the mouse may not be a suitable model to study the pathophysiology of the intronic *CEP290* c.2991+1655AG mutation, due to differential recognition of splice sites between humans and mice. Together, our results emphasize that caution is warranted when generating animal models for human genetic diseases caused by splice mutations. 

## Supporting Information

Table S1 **Primer sequences.**
(DOC)

## References

[1] Koenekoop RK (2004) An overview of Leber congenital amaurosis: a model to understand human retinal development. Surv Ophthalmol 49: 379-398. doi:10.1016/j.survophthal.2004.04.003. PubMed: 15231395. 15231395

[2] Stone EM (2007) Leber congenital amaurosis - a model for efficient genetic testing of heterogeneous disorders: LXIV Edward Jackson Memorial Lecture. Am J Ophthalmol 144: 791-811. doi:10.1016/j.ajo.2007.08.022. PubMed: 17964524. 17964524

[3] den Hollander AI, Roepman R, Koenekoop RK, Cremers FPM (2008) Leber congenital amaurosis: genes, proteins and disease mechanisms. Prog Retin Eye Res 27: 391-419. doi:10.1016/j.preteyeres.2008.05.003. PubMed: 18632300. 18632300

[4] Coppieters F, Lefever S, Leroy BP, De Baere E (2010) CEP290, a gene with many faces: mutation overview and presentation of CEP290base. Hum Mutat 31: 1097-1108. doi:10.1002/humu.21337. PubMed: 20690115. 20690115

[5] den Hollander AI, Koenekoop RK, Yzer S, Lopez I, Arends ML et al. (2006) Mutations in the CEP290 (NPHP6) gene are a frequent cause of Leber congenital amaurosis. Am J Hum Genet 79: 556-561. doi:10.1086/507318. PubMed: 16909394. 16909394 PMC1559533

[6] Perrault I, Delphin N, Hanein S, Gerber S, Dufier JL et al. (2007) Spectrum of NPHP6/CEP290 mutations in Leber congenital amaurosis and delineation of the associated phenotype. Hum Mutat 28: 416. doi:10.1002/humu.9485. PubMed: 17345604. 17345604

[7] Littink KW, Pott JW, Collin RWJ, Kroes HY, Verheij JB et al. (2010) A novel nonsense mutation in CEP290 induces exon skipping and leads to a relatively mild retinal phenotype. Invest Ophthalmol Vis Sci 51: 3646-3652. doi:10.1167/iovs.09-5074. PubMed: 20130272. 20130272

[8] Hildebrandt F, Zhou W (2007) Nephronophthisis-associated ciliopathies. J Am Soc Nephrol 18: 1855-1871. doi:10.1681/ASN.2006121344. PubMed: 17513324. 17513324

[9] Sayer JA, Otto EA, O'Toole JF, Nurnberg G, Kennedy MA et al. (2006) The centrosomal protein nephrocystin-6 is mutated in Joubert syndrome and activates transcription factor ATF4. Nat Genet 38: 674-681. doi:10.1038/ng1786. PubMed: 16682973. 16682973

[10] Leitch CC, Zaghloul NA, Davis EE, Stoetzel C, Diaz-Font A et al. (2008) Hypomorphic mutations in syndromic encephalocele genes are associated with Bardet-Biedl syndrome. Nat Genet 40: 443-448. doi:10.1038/ng.97. PubMed: 18327255. 18327255

[11] Frank V, den Hollander AI, Brüchle NO, Zonneveld MN, Nürnberg G et al. (2008) Mutations of the CEP290 gene encoding a centrosomal protein cause Meckel-Gruber syndrome. Hum Mutat 29: 45-52. doi:10.1002/humu.20614. PubMed: 17705300. 17705300

[12] Nagase T, Ishikawa K, Nakajima D, Ohira M, Seki N et al. (1997) Prediction of the coding sequences of unidentified human genes. VII. The complete sequences of 100 new cDNA clones from brain which can code for large proteins in vitro. DNA Res 4: 141-150. doi:10.1093/dnares/4.2.141. PubMed: 9205841. 9205841

[13] Craige B, Tsao CC, Diener DR, Hou Y, Lechtreck KF et al. (2010) CEP290 tethers flagellar transition zone microtubules to the membrane and regulates flagellar protein content. J Cell Biol 190: 927-940. doi:10.1083/jcb.201006105. PubMed: 20819941. 20819941 PMC2935561

[14] Garcia-Gonzalo FR, Corbit KC, Sirerol-Piquer MS, Ramaswami G, Otto EA et al. (2011) A transition zone complex regulates mammalian ciliogenesis and ciliary membrane composition. Nat Genet 43: 776-784. doi:10.1038/ng.891. PubMed: 21725307. 21725307 PMC3145011

[15] Barbelanne M, Song J, Ahmadzai M, Tsang WY (2013) Pathogenic NPHP5 mutations impair protein interaction with Cep290, a prerequisite for ciliogenesis. Hum: Mol Genet. 10.1093/hmg/ddt100 PMC3797088 23446637

[16] Chang B, Khanna H, Hawes N, Jimeno D, He S et al. (2006) In-frame deletion in a novel centrosomal/ciliary protein CEP290/NPHP6 perturbs its interaction with RPGR and results in early-onset retinal degeneration in the rd16 mouse. Hum Mol Genet 15: 1847-1857. doi:10.1093/hmg/ddl107. PubMed: 16632484. 16632484 PMC1592550

[17] Estrada-Cuzcano A, Roepman R, Cremers FPM, den Hollander AI, Mans DA (2012) Non-syndromic retinal ciliopathies: translating gene discovery into therapy. Hum Mol Genet 21: R111-R124. doi:10.1093/hmg/dds298. PubMed: 22843501. 22843501

[18] Kolb H (2003) How the Retina Works. Am Sci 91: 28-35. doi:10.1511/2003.1.28.

[19] Menotti-Raymond M, David VA, Schäffer AA, Stephens R, Wells D et al. (2007) Mutation in CEP290 discovered for cat model of human retinal degeneration. J Hered 98: 211-220. doi:10.1093/jhered/esm019. PubMed: 17507457. 17507457

[20] Narfström K (1985) Progressive retinal atrophy in the Abyssinian cat. Clinical characteristics. Invest Ophthalmol Vis Sci 26: 193-200. PubMed: 3972501. 3972501

[21] Rasband WS (1997-2012) Image J. In: Maryland USA, U. S. National Institutes of Health B. Available: http://imagej.nih.gov/ij/. Accessed 2013 September 22.

[22] Collin RWJ, den Hollander AI, van der Velde-Visser SD, Bennicelli J, Bennett J et al. (2012) Antisense Oligonucleotide (AON)-based Therapy for Leber Congenital Amaurosis Caused by a Frequent Mutation in CEP290. Mol Ther Nucleic Acids 1: e14. doi:10.1038/mtna.2012.3. PubMed: 23343883. 23343883 PMC3381589

[23] Chang B, Hawes NL, Hurd RE, Wang J, Howell D et al. (2005) Mouse models of ocular diseases. Vis Neurosci 22: 587-593. PubMed: 16332269. 16332269 10.1017/S0952523805225075

[24] Won J, Shi LY, Hicks W, Wang J, Hurd R et al. (2011) Mouse model resources for vision research. J Ophthalmol, 2011: 2011: 391384. PubMed: 21052544 10.1155/2011/391384 PMC2968714 21052544

[25] Song BJ, Tsang SH, Lin C-S (2007) Genetic models of retinal degeneration and targets for gene therapy. Gene Therapy Molecular Biol 11: 229-262.

[26] Gerard X, Perrault I, Hanein S, Silva E, Bigot K et al. (2012) AON-mediated Exon Skipping Restores Ciliation in Fibroblasts Harboring the Common Leber Congenital Amaurosis CEP290 Mutation. Mol Ther Nucleic Acids 1: e29. doi:10.1038/mtna.2012.21. PubMed: 23344081. 23344081 PMC3390222

[27] Gladman JT, Bebee TW, Edwards C, Wang X, Sahenk Z et al. (2010) A humanized Smn gene containing the SMN2 nucleotide alteration in exon 7 mimics SMN2 splicing and the SMA disease phenotype. Hum Mol Genet 19: 4239-4252. doi:10.1093/hmg/ddq343. PubMed: 20705738. 20705738 PMC2951869

[28] Hims MM, Shetty RS, Pickel J, Mull J, Leyne M et al. (2007) A humanized IKBKAP transgenic mouse models a tissue-specific human splicing defect. Genomics 90: 389-396. doi:10.1016/j.ygeno.2007.05.012. PubMed: 17644305. 17644305 PMC1976430

[29] Vadolas J, Nefedov M, Wardan H, Mansooriderakshan S, Voullaire L et al. (2006) Humanized beta-thalassemia mouse model containing the common IVSI-110 splicing mutation. J Biol Chem 281: 7399-7405. doi:10.1074/jbc.M512931200. PubMed: 16421096. 16421096

[30] Yang Y, Swaminathan S, Martin BK, Sharan SK (2003) Aberrant splicing induced by missense mutations in BRCA1: clues from a humanized mouse model. Hum Mol Genet 12: 2121-2131. doi:10.1093/hmg/ddg222. PubMed: 12915465. 12915465

[31] Garanto A, Riera M, Pomares E, Permanyer J, de Castro-Miró M et al. (2011) High transcriptional complexity of the retinitis pigmentosa CERKL gene in human and mouse. Invest Ophthalmol Vis Sci 52: 5202-5214. doi:10.1167/iovs.10-7101. PubMed: 21508105. 21508105

[32] Roca X, Krainer AR, Eperon IC (2013) Pick one, but be quick: 5' splice sites and the problems of too many choices. Genes Dev 27: 129-144. doi:10.1101/gad.209759.112. PubMed: 23348838. 23348838 PMC3566305

[33] Collin RWJ, Littink KW, Klevering BJ, van den Born LI, Koenekoop RK et al. (2008) Identification of a 2 Mb human ortholog of Drosophila eyes shut/spacemaker that is mutated in patients with retinitis pigmentosa. Am J Hum Genet 83: 594-603. doi:10.1016/j.ajhg.2008.10.014. PubMed: 18976725. 18976725 PMC2668042

[34] Abd El-Aziz MM, Barragan I, O'Driscoll CA, Goodstadt L, Prigmore E et al. (2008) EYS, encoding an ortholog of Drosophila spacemaker, is mutated in autosomal recessive retinitis pigmentosa. Nat Genet 40: 1285-1287. doi:10.1038/ng.241. PubMed: 18836446. 18836446 PMC2719291

[35] Howes KA, Pennesi ME, Sokal I, Church-Kopish J, Schmidt B et al. (2002) GCAP1 rescues rod photoreceptor response in GCAP1/GCAP2 knockout mice. EMBO J 21: 1545-1554. doi:10.1093/emboj/21.7.1545. PubMed: 11927539. 11927539 PMC125366

[36] Makino CL, Peshenko IV, Wen XH, Olshevskaya EV, Barrett R et al. (2008) A role for GCAP2 in regulating the photoresponse. Guanylyl cyclase activation and rod electrophysiology in GUCA1B knock-out mice. J Biol Chem 283: 29135-29143. doi:10.1074/jbc.M804445200. PubMed: 18723510. 18723510 PMC2570858

[37] Garanto A, Vicente-Tejedor J, Riera M, de la Villa P, Gonzàlez-Duarte R et al. (2012) Targeted knockdown of Cerkl, a retinal dystrophy gene, causes mild affectation of the retinal ganglion cell layer. Biochim Biophys Acta 1822: 1258-1269. doi:10.1016/j.bbadis.2012.04.004. PubMed: 22549043. 22549043

[38] Liu X, Bulgakov OV, Darrow KN, Pawlyk B, Adamian M et al. (2007) Usherin is required for maintenance of retinal photoreceptors and normal development of cochlear hair cells. Proc Natl Acad Sci U S A 104: 4413-4418. doi:10.1073/pnas.0610950104. PubMed: 17360538. 17360538 PMC1838616

[39] Kurth I, Thompson DA, Rüther K, Feathers KL, Chrispell JD et al. (2007) Targeted disruption of the murine retinal dehydrogenase gene Rdh12 does not limit visual cycle function. Mol Cell Biol 27: 1370-1379. doi:10.1128/MCB.01486-06. PubMed: 17130236. 17130236 PMC1800705

[40] Acland GM, Aguirre GD, Ray J, Zhang Q, Aleman TS et al. (2001) Gene therapy restores vision in a canine model of childhood blindness. Nat Genet 28: 92-95. doi:10.1038/ng0501-92. PubMed: 11326284. 11326284

