# Supplementary material for: Unexpected CEP290 mRNA Splicing in a Humanized Knock-In Mouse Model for Leber Congenital Amaurosis
Source: PLoS One. 2013 Nov 6;8(11):e79369. doi: 10.1371/journal.pone.0079369 (PMC3819269; doi:10.1371/journal.pone.0079369)
Supplement: Table S1 — Primer sequences. (DOC) [file pone.0079369.s001.doc]

**Table S1 – Primer sequences**

| **Forward oligonucleotides** | | **Reverse oligonucleotides** | |
| --- | --- | --- | --- |
| **Name** | **Sequence 5’→3’** | **Name** | **Sequence 5’→3’** |
| A | ACGCAAAAGTGGAAGAGTGG | B | TCGGCCTCTTTTGTTTTCTC |
| C | GACGTCATAGCCATGGAAGC | D | TGACAGTGTTCAGCCCTCTG |
| E | TTTCAAGATTGCAGCTCTCCAAAAAGTCG | F | ACTCCACTTGTTCTTTTAAGGAGATGTTT |
| ActF | ACTGGGACGACATGGAGAAG | ActR | TCTCAGCTGTGGTGGTGAAG |
| G | GCACCTGGCCCCAGTTG | I | AGACTCCACTTGTTCTTTTAAGGAG |
| H | CATAGCTCATTGCAGCCTTG |  |  |
